# The Effect of *Ipomoea batatas* on Glycemic Control and Lipid Profiles in Animal Models: A Systematic Review and Meta‐Analysis

**DOI:** 10.1155/ijfo/1713161

**Published:** 2026-06-15

**Authors:** Hapsari Sulistya Kusuma, Mohammad Sulchan, Heri-Nugroho H. S., Suhartono Suhartono, Diana Nur Afifah, Endang Mahati, Arimi Fitri Mat Ludin, Adriyan Pramono

**Affiliations:** ^1^ Doctoral Study Program of Medicine and Health Science, Universitas Diponegoro, Semarang, Indonesia, undip.ac.id; ^2^ Nutrition Study Program, Faculty of Health and Nursing Sciences, Universitas Muhammadiyah, Semarang, Indonesia; ^3^ Department of Nutrition Science, Faculty of Medicine, Universitas Diponegoro, Semarang, Indonesia, undip.ac.id; ^4^ Division of Endocrinology, Department of Internal Medicine, Faculty of Medicine, Universitas Diponegoro, Semarang, Indonesia, undip.ac.id; ^5^ Department of Environmental Health, Faculty of Public Health, Universitas Diponegoro, Semarang, Indonesia, undip.ac.id; ^6^ Center of Nutrition Research (CENURE), Faculty of Medicine, Universitas Diponegoro, Semarang, Indonesia, undip.ac.id; ^7^ Department of Pharmacology and Therapy, Faculty of Medicine, Universitas Diponegoro, Semarang, Indonesia, undip.ac.id; ^8^ Center for Biomedical Research (CEBIOR), Faculty of Medicine, Universitas Diponegoro, Semarang, Indonesia, undip.ac.id; ^9^ Centre for Healthy Aging and Wellness, Faculty of Health Sciences, Universiti Kebangsaan Malaysia, Kuala Lumpur, Malaysia, ukm.my

**Keywords:** antioxidant, dietary fiber, dyslipidemia, experimental model, glycemic index, insulin resistance, sweet potato

## Abstract

**Background:**

Sweet potato (*Ipomoea batatas*), a fiber‐rich food with a low glycemic index, has shown potential in regulating glucose and lipid metabolism. This systematic review and meta‐analysis aimed to evaluate its effects on glycemic and lipid parameters in animal models of obesity and insulin resistance.

**Methods:**

Relevant experimental studies published in English were identified through PubMed, Scopus, Cochrane Library, and Embase, excluding in silico models. Data were extracted for fasting glucose, total cholesterol, triglycerides, low‐density lipoprotein cholesterol (LDL‐C), and high‐density lipoprotein cholesterol (HDL‐C). Standardized mean differences (SMDs) with 95% confidence intervals were calculated, and risk of bias (RoB) was assessed using the SYRCLE RoB tool. The review adhered to the Preferred Reporting Items for Systematic Reviews and Meta‐Analyses (PRISMA) guidelines and was registered in PROSPERO (CRD42025599301). The literature search was conducted from November 2024 to August 2025.

**Results:**

Ten studies involving 428 animals were included in the meta‐analysis. The meta‐analysis demonstrated that *I. batatas* administration significantly reduced fasting plasma glucose (SMD = −3.88; 95% CI: −5.63 to −2.13; *p* < 0.0001; *I* ^2^ = 87*%*) and improved lipid parameters, including reductions in total cholesterol, triglycerides, and LDL‐C and an increase in HDL‐C (SMD total cholesterol −21.5 [−38.49 to −4.55]; *p* = 0.01; *I* ^2^ = 93*%*; SMD triglycerides −4.83 [−9.07 to −0.60]; *p* = 0.03; *I* ^2^ = 93*%*; SMD LDL‐C −9.86 [−19.21 to −0.50]; *p* = 0.04; *I* ^2^ = 96*%*; SMD HDL‐C 11.13 [2.06 to 20.21]; *p* = 0.02; *I* ^2^ = 95*%*). These effects were observed for both leaf and root extracts.

**Conclusion:**

Supplementation with *I. batatas* favorably influences fasting glucose and lipid profiles in animal models, suggesting its potential as a functional dietary component for improving metabolic health.

## 1. Introduction

The sweet potato (*Ipomoea batatas*) has potential implications for metabolic health, particularly in addressing insulin resistance. Insulin resistance is commonly associated with central obesity. The mechanism begins when adipose tissue stores lipids as triacylglycerols from chylomicron absorption and periodically releases nonesterified fatty acids to meet increasing energy demands. When energy intake exceeds expenditure, fatty acids accumulate beyond the adipose tissue storage capacity, leading to dysfunction. This dysfunction impairs insulin secretion, disrupting its role in inhibiting triacylglycerol extraction and endogenous lipolysis. As a result, insulin secretion increases, leading to higher circulating insulin levels. Without sufficient lipid buffering capacity, fatty acids enter the bloodstream and accumulate in the muscle, liver, and pancreas, contributing to insulin resistance [1].

Research on the effects of diets on insulin resistance in experimental animals and humans has been conducted; however, it has yielded less effective and practical results. A study by Utari et al. [2] indicated that low‐calorie and low‐glycemic index (GI) diets were less effective in reducing insulin resistance based on the HOMA‐IR index in obesity, due to the lack of proper intake monitoring. Despite this, a balanced, low‐calorie, and low‐glycemic dietary approach has been shown to result in significant weight loss, which is beneficial for treating obesity. Consequently, dietary therapy, especially with proper intake monitoring, remains a promising approach to manage insulin resistance, a common consequence of obesity.

Current research focuses on identifying alternative staple food ingredients to replace rice. Foods high in fiber and low in GI are considered ideal for individuals at risk of insulin resistance. Based on this, *I. batatas* is a promising candidate due to its high fiber content, low GI of 46, and bioactive compounds like *β*‐carotene, which are absent in rice. Compared to rice (GI of 79.6), sweet potato has one‐third the calorie content, has 10 times more fiber, and contains *β*‐carotene, which has antidiabetic potential [3–5].

A review of the literature revealed limited in vivo and in vitro studies on *I. batatas* in relation to glycemic control and lipid profiles. Notably, Naomi et al. [6] focused primarily on diabetic retinopathy, examining retinal protection rather than systemic glycemic control or lipid profile modulation. Furthermore, this study did not specifically address Type 2 diabetes mellitus, limiting its applicability to the broader context of insulin resistance.

Despite these limitations, there is no comprehensive synthesis addressing the effects of *I. batatas* on glycemic control and lipid profiles in animal models. Existing studies are heterogeneous in design and often focus on specific disease outcomes rather than broader metabolic parameters related to insulin resistance. Further investigation is needed to explore the impact of sweet potato intake on central obesity, particularly its effects on glycemic control and lipid regulation, which contribute to insulin resistance.

Therefore, this study was designed to systematically review and meta‐analyze the effects of sweet potato on glycemic control and lipid profiles in animal models. This approach enables a comprehensive synthesis of the available evidence and is based on preliminary yet promising findings regarding its dietary effects ([7, 8]).

## 2. Methods

### 2.1. Overview of Reviews

This systematic review and meta‐analysis (SR‐MA) followed the Preferred Reporting Items for Systematic Reviews and Meta‐Analyses (PRISMA) 2020 guidelines ([9]) and was prospectively registered in the PROSPERO database (CRD42025599301). The PRISMA 2020 checklist is provided in Supporting Information 2: File S1 and Supporting Information 3: File S2.

### 2.2. Search Strategy

A comprehensive search was conducted across five databases: PubMed/MEDLINE, Scopus, Cochrane Library, EBSCO, and ProQuest. Studies published between January 1, 2000, and March 21, 2025, were included, using combinations of the terms “*Ipomoea batatas*,” “glycemic control,” “glucose homeostasis,” “lipid profile,” and “meta‐analysis.” Only intervention studies published in English and conducted using rodent models were included. Reference lists of eligible articles were screened to identify additional relevant studies. The search was conducted from November 2024 to August 2025.

The same keyword strategy was applied across all databases using Boolean operators (“AND” and “OR”). The core search string was as follows: (“*Ipomoea batatas*” OR “sweet potato” OR “sweet potato extract”) AND (“type 2 diabetes” OR “blood glucose” OR “insulin resistance” OR “hyperglycemia” OR “glycemic control”) AND (“lipid metabolism” OR “lipid profile” OR “cholesterol” OR “triglycerides” OR “dyslipidemia”) AND (“animal study” OR “animal model” OR “in vivo” OR “rodent model” OR “experimental model”). The complete search strategy for PubMed is provided in Supporting Information 4: Table S1, in accordance with PRISMA 2020 recommendations.

To minimize publication bias, additional gray literature searches were conducted in the WHO database, Universitas Diponegoro (UNDIP) institutional repository, and Indonesian Garuda database using the keywords “sweet potato,” “*Ipomoea batatas*,” “glucose,” “rats,” and “lipid.” No eligible records were identified in the WHO and UNDIP databases. Four records retrieved from the Garuda database were excluded because they did not meet the inclusion criteria.

### 2.3. Study Selection, Inclusion, and Exclusion

Two reviewers independently screened titles and abstracts from the databases and additional sources to identify eligible studies. Interrater reliability was assessed using Cohen’s kappa coefficient, indicating moderate agreement (*κ* = 0.57). Articles were included if they met the following criteria: (1) randomized controlled animal studies evaluating *I. batatas* supplementation; (2) use of rat and/or mouse models representing obesity, metabolic syndrome, or Type 2 diabetes; and (3) measurable outcomes related to glycemic or lipid parameters. There were no restrictions regarding dose, frequency, or duration of *I. batatas* administration.

Studies were excluded if they (1) were observational or noninterventional; (2) used in vitro, ex vivo, or in silico models; (3) involved animals receiving additional supplements unrelated to *I. batatas*; or (4) failed to report relevant glycemic or lipid outcomes. Full texts of shortlisted papers were independently reviewed by two authors, with disagreements resolved through discussion with a third reviewer.

### 2.4. Data Extraction and Synthesis

Data extraction was performed independently by two authors using predesigned Microsoft Excel spreadsheets to ensure consistency and completeness. Discrepancies in extracted data were discussed and resolved by consensus. Extracted information included study design, intervention details, sample characteristics, and quantitative outcomes. If essential information was unavailable or unclear, no attempts were made to contact original study authors. For each included study, details were extracted on the publication reference, study design, animal characteristics (species, sex, and disease model), intervention description (form, dosage, and duration of *I. batatas*), comparator groups, and reported outcomes. Outcomes included fasting plasma glucose (FPG), oral glucose tolerance (2‐h OGTT), HOMA‐IR, and lipid measures (triglycerides, total cholesterol, low‐density lipoprotein cholesterol [LDL‐C], and high‐density lipoprotein cholesterol [HDL‐C]). Data expressed in nmol/L were standardized to mg/dL. All continuous variables were summarized as standardized mean differences (SMDs) with 95% confidence intervals.

Key conclusions from the authors were also extracted to contextualize findings. The quality of each study was assessed, and effect sizes were interpreted using standard conventions: SMDs of > 0.8 (large), 0.5–0.8 (moderate), 0.2–0.5 (small), and < 0.2 (minimal). Potential overlap among studies was checked to ensure data independence.

SR‐MAs were conducted using a random‐effects model to account for between‐study variability. Statistical heterogeneity was assessed using the *I* ^2^ statistic. Sensitivity analyses were performed using a leave‐one‐out approach to assess the robustness of the pooled estimates. Publication bias (small‐study effects) was assessed through visual inspection of funnel plots. Although 10 studies were included, formal statistical tests for funnel plot asymmetry (e.g., Egger’s regression test) were not performed due to limited reliability in meta‐analyses with a small number of studies.

### 2.5. Quality Assessment

The methodological quality of included studies was assessed using the SYRCLE risk‐of‐bias (RoB) tool, specifically designed for animal intervention studies and adapted from the Cochrane RoB tool. The SYRCLE RoB tool includes 10 domains, covering potential sources of bias at different study stages, including selection, performance, detection, attrition, and reporting bias. The 10 domains assessed include the following: (1) sequence generation (randomization), (2) baseline characteristics, (3) allocation concealment, (4) random housing, (5) blinding of caregivers and/or investigators, (6) random outcome assessment, (7) blinding of outcome assessors, (8) incomplete outcome data, (9) selective outcome reporting, and (10) other sources of bias.

Overall study quality was categorized as “good,” “fair,” or “poor,” based on the number of unmet quality criteria (0, 1, or ≥ 2, respectively). Assessments were performed independently by two reviewers. The original quality ratings from the included studies were documented for comparison.

## 3. Results and Discussion

### 3.1. Overview of Reviews

**Figure 1:**
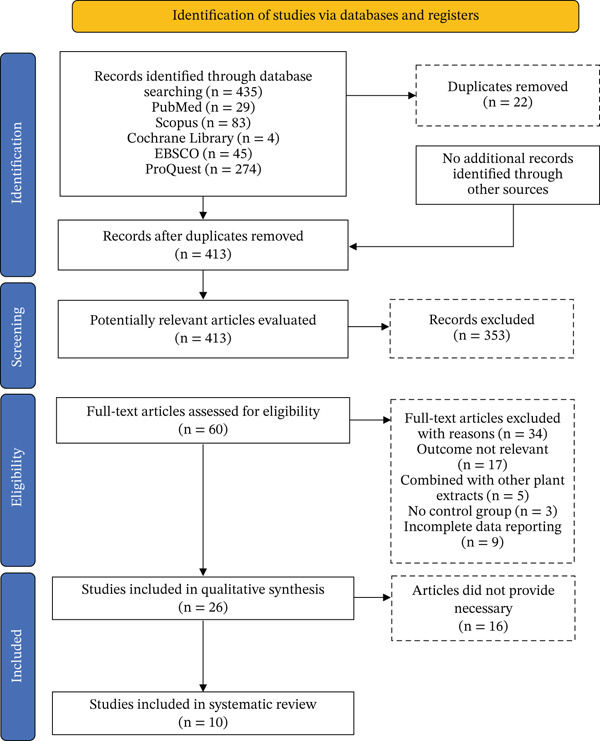
PRISMA flow diagram depicting the screening process for inclusion of studies in the qualitative synthesis. Abbreviation: PRISMA, Preferred Reporting Items for Systematic Reviews and Meta‐Analyses.

A total of 435 records were initially identified through database searches. After removing duplicates, 413 unique articles remained. Screening titles and abstracts excluded 183 papers that did not meet the inclusion criteria. Sixty full‐text articles were assessed for eligibility, with 26 studies retained for qualitative synthesis. Ten original studies met all criteria and were included in the quantitative meta‐analysis (Figure 1 and Table 1).

**Table 1:** Characteristics of included systematic reviews.

Reference	Population characteristics	Intervention and comparison	Outcomes	Number of participants
[10]	HFD‐induced hyperglycemia, various doses of purple leaf	NS: normal rats + normal saline DNS: diabetic rats + normal saline D25G: diabetic rats + gliclazide 25 mg/kg DT 200: diabetic rats + PSPL extract 200 mg/kg DT 400: diabetic rats + PSPL extract 400 mg/kg	FBG, TAC/FRAP, IL‐17A	50 (10/group)
[11]	T2DM mice (model induced), SPLP‐L and SPLP‐H groups	NC: normal control group DC: diabetic control group MET: metformin 100 mg/kg BW SPLP‐L: low‐dose sweet potato leaf polyphenols (SPLP) (80 mg/kg BW) SPLP‐H: high‐dose SPLP (150 mg/kg BW) ETH‐L: low‐dose ethyl acetate extract (ETH) (10 mg/kg BW) ETH‐H: high‐dose ETH (30 mg/kg BW) KAE‐L: low‐dose kaempferol (KAE) (10 mg/kg BW) KAE‐H: high‐dose KAE (30 mg/kg BW)	FBG, AUC, insulin	90 (10/group)
[12]	Sprague‐Dawley rats, male, aged 2–3 months, 180–200 g body weight, Type 1 diabetes model induced by multiple low‐dose streptozotocin (STZ) injections (40 mg/kg BW) intraperitoneally for 5 consecutive days. Diabetes was confirmed when FBG was > 200 mg/dL	IG1 (0.25 g/kg): *Ipomoea batatas* leaf extract (intervention group) IG2 (0.8 g/kg): *Ipomoea batatas* leaf extract (intervention group) IG3 (2.5 g/kg): *Ipomoea batatas* leaf extract (intervention group) NC (normal control): no treatment DC (diabetic control): STZ‐induced diabetic rats (untreated) ↓ FBG, ↓ insulitis severity, ↑ *β*‐cell insulin expression	FBG, insulitis severity, *β*‐cell insulin expression	25 (5/group)
[13]	STZ‐induced diabetic rats, treated with flavonoid extract	NC: normal control group DC: diabetic control group DLF: diabetic + FIBL (50 mg/kg) DMF: diabetic + FIBL (100 mg/kg) DHF: diabetic + FIBL (150 mg/kg) DG: diabetic + glibenclamide (0.25 mg/kg)	FBG, lipids, HDL	60 (10/group)
[14]	Hypercholesterolemic rats by diet, 1% cholesterol	E400: *Ipomoea batatas* leaf extract (400 mg/kg) E500: *Ipomoea batatas* leaf extract (500 mg/kg) E600: *Ipomoea batatas* leaf extract (600 mg/kg) NC: normocholesterolemic group HC: hypercholesterolemic control group	Lipids, histological improvement	50 (10/group)
[15]	Wistar rats, fed high‐cholesterol diet	Control group: high‐cholesterol diet without supplementation PSPE group: high‐cholesterol diet with purple sweet potato extract at 200 mg/day/rat	TC, TG, LDL, MDA, HDL, SOD, Apo‐E, netrin‐1	20 (10/group)
[16]	Male mice (*Mus musculus*), aged 12–14 weeks, 25–30 g body weight, diabetes induced by a single intraperitoneal injection of STZ (60 mg/kg BW) plus a 10% dextrose diet to promote insulin resistance	PSP‐CMC: purple sweet potato extract loaded with CMC‐alginate nanocapsules (0.5 mL) PSP: regular purple sweet potato extract (0.5 mL) POS: positive control (glibenclamide 0.39 mg/L, 1 mL) NEG: negative control (0.5% CMC sodium placebo)	FBG	24 (6/group)
[17]	KK‐Ay mice, spontaneous T2DM model, starting age 4 weeks	CG (control group): no treatment, just fed normal diet SP (sweet potato leaf extract group): 300 mg/kg of leaf extract administered daily for 5 weeks	FBG, insulin	16 (8/group)
[18]	Male rats, cholesterol‐enriched diet induced hyperlipidemia	CG: control group PC Lam extract: 0.4 cc/rat/day IB L tablet: 200 mg/rat/day PC Lam extract + IB L tablet: 0.4 cc + 200 mg/rat/day	TC, TG, LDL, HDL, MDA, SOD	28 (7/group)
[19]	Male Kunming mice, 8 weeks old, 18–20 g, T2DM induced with HFD + STZ	Control group (CG): standard diet + vehicle Model group (MG): HFD + STZ + vehicle LP group (LP): HFD + STZ + low‐dose PSPA (227.5 mg/kg BW) MP group (MP): HFD + STZ + medium‐dose PSPA (455 mg/kg BW) HP group (HP): HFD + STZ + high‐dose PSPA (910 mg/kg BW)	FBG, HbA1c, GHB, TNF‐*α*, IL‐6, LPS, GSH‐PX	65 (13/group)

### 3.2. Characteristics of Included Studies

The studies included in the meta‐analysis are summarized in Table 1. The included studies spanned from 2015 to 2025, all of which were randomized controlled trials (*n* = 10). The intervention period for *I. batatas* administration ranged from 1 to 12 weeks. Six studies investigated leaf extracts, whereas four evaluated root extracts. All trials reported pre‐ and postintervention outcomes measured at the end of each treatment period.

### 3.3. Quality of Original Studies Included in the SR‐MA

Based on SYRCLE criteria, one study was classified as “good” quality and nine as “fair.” None were rated as “poor.” All included studies clearly defined their research questions and eligibility criteria, applied systematic search strategies, and comprehensively reported study characteristics and findings.

### 3.4. Effectiveness of *I. batatas*

#### 3.4.1. Glycemic Control

**Figure 2:**
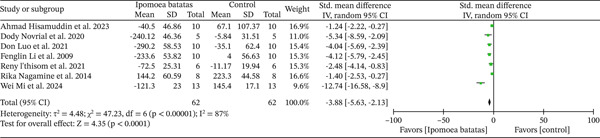
Overview of reviews on the effect of *Ipomoea batatas* on fasting blood glucose.

Seven studies reported a significant decrease in fasting blood glucose (FBG) among animals receiving *I. batatas* compared to the control group, regardless of whether the extract was derived from leaves or roots. The SMD for FBG reduction was −3.88 [−5.63 to −2.13], with *p* < 0.0001 and *I* ^2^ = 87*%* (Figure 2). These decreases in blood glucose highlight effective glycemic control and prompt further investigation into the underlying mechanisms.

One mechanism involves the relatively low GI of *I. batatas*. Numerous studies have explored the role of *I. batatas* in regulating FBG, particularly in the context of diabetes. Research shows that sweet potatoes have a lower GI compared to other high‐carbohydrate foods, making them beneficial in managing Type 2 diabetes by reducing FBG spikes and the overall glycemic response [20–22].

In addition to their low GI, the dietary fiber in sweet potatoes plays a key role in maintaining glucose homeostasis. The dietary fiber in sweet potatoes contributes to lower FBG levels and improved glycemic control by delaying carbohydrate absorption. Evidence suggests that the combined benefits of low‐glycemic‐load meals and fiber improve FBG and other diabetes‐related indicators ([23], [21, 24]).

Incorporating low‐GI foods, such as sweet potatoes, into daily meals can improve glycemic management, consistent with meta‐analysis findings showing a significant reduction in FPG among individuals following low‐GI diets [20–22].

Our meta‐analyses included studies on *I. batatas* in diverse forms, including both leaf and root extracts. Subgroup analyses indicated that both sweet potato leaf and root interventions significantly reduce FBG (Supporting Information 1: Figure S1).

#### 3.4.2. Lipid Profiles

**Figure 3:**
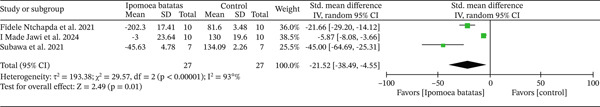
Overview of reviews on the effect of *Ipomoea batatas* on total cholesterol.

**Figure 4:**
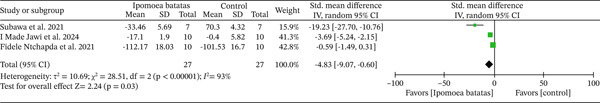
Overview of reviews on the effect of *Ipomoea batatas* on triglycerides.

**Figure 5:**
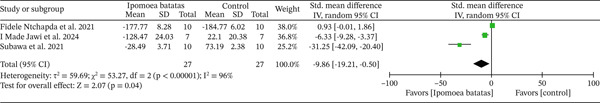
Overview of reviews on the effect of *Ipomoea batatas* on LDL‐C.

**Figure 6:**
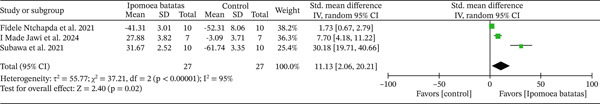
Overview of reviews on the effect of *Ipomoea batatas* on HDL‐C.

Three of 10 studies reported significant decreases in total cholesterol, triglycerides, and LDL‐C, along with an increase in HDL‐C, in the *I. batatas* intervention group compared to the control group, regardless of whether the extract was derived from leaves or roots. The SMDs for lipid parameters were as follows: total cholesterol −21.5 [−38.49 to −4.55], *p* = 0.01; triglycerides −4.83 [−9.07 to −0.60], *p* = 0.03; LDL‐C −9.86 [−19.21 to −0.50], *p* = 0.04; HDL‐C 11.13 [2.06 to 20.21], *p* = 0.02 (Figures 3, 4, 5, and 6).

Although the pooled analysis showed statistically significant improvements in FPG levels and lipid parameters, the clinical significance of these findings must be interpreted cautiously. The use of SMDs limits direct clinical interpretation, as effect sizes are expressed in unitless values rather than conventional clinical measures (e.g., mg/dL). The high heterogeneity observed across studies (*I* ^2^ > 85*%*) indicates substantial variability in intervention type, dosage, duration, and animal models, which may have contributed to the variability in pooled effects. Therefore, these findings should be regarded as evidence of potential biological efficacy in animal models rather than direct therapeutic effects in humans.

### 3.5. Publication Bias

**Figure 7:**
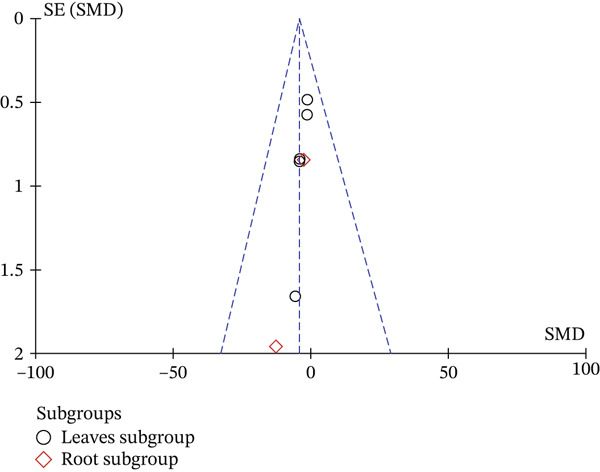
Funnel plot of the effect of *Ipomoea batatas* on fasting blood glucose.

**Figure 8:**
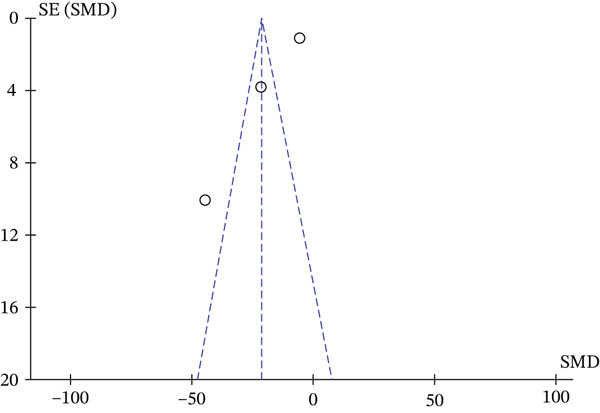
Funnel plot of the effect of *Ipomoea batatas* on total cholesterol.

**Figure 9:**
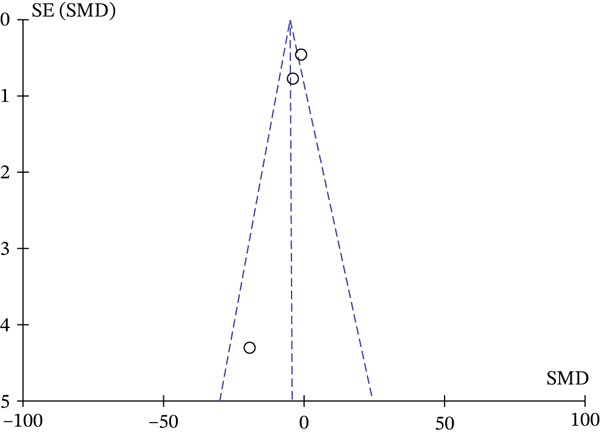
Funnel plot of the effect of *Ipomoea batatas* on triglycerides.

**Figure 10:**
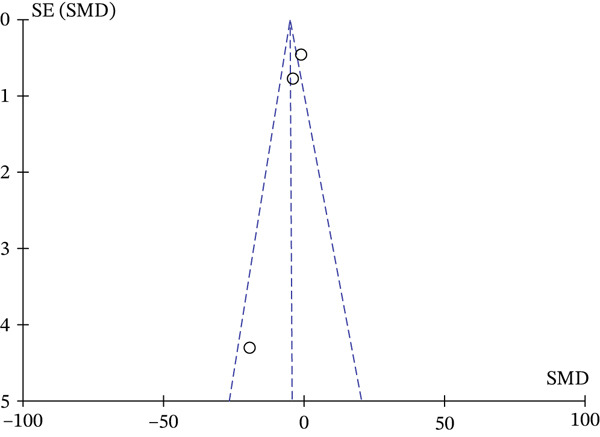
Funnel plot of the effect of *Ipomoea batatas* on LDL‐C.

**Figure 11:**
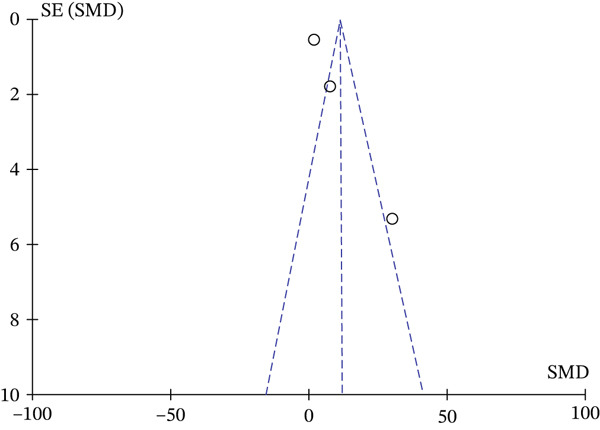
Funnel plot of the effect of *Ipomoea batatas* on HDL‐C.

Publication bias was assessed through visual inspection of funnel plots for each outcome, including FBG (Figure 7), total cholesterol (Figure 8), triglycerides (Figure 9), LDL‐C (Figure 10), and HDL‐C (Figure 11). The funnel plots appeared relatively symmetrical, suggesting no substantial evidence of publication bias among the included studies. However, these findings should be interpreted cautiously due to the limited number of studies for each outcome (≤ 10).

Interest in *I. batatas* has grown due to its potential impact on lipid profiles. The unique nutritional composition, including high dietary fiber, antioxidants, and other bioactive compounds, may positively influence lipid metabolism. The dietary fiber in *I. batatas* contributes to lower FBG levels and improved glycemic control by delaying carbohydrate absorption. Evidence suggests that the combined benefits of low‐glycemic‐load meals and fiber improve FBG and other diabetes‐related indicators [25, 26].

The antioxidant activity in *I. batatas* roots may help reduce oxidative stress, which is associated with lipid profile deterioration and cardiovascular risk factors. Studies suggest that regular consumption of antioxidant‐rich foods can reduce triglycerides and increase HDL‐C levels [27, 28].

Sweet potatoes, with their relatively low GI, raise blood sugar levels gradually and may improve lipid metabolism by enhancing insulin sensitivity. Since insulin regulates lipid synthesis and breakdown, increased insulin sensitivity is often associated with improved lipid profiles [27, 29]. This relationship underscores the importance of incorporating low‐glycemic foods, such as sweet potatoes, in managing metabolic syndrome and dyslipidemia to improve cardiovascular health.

Additionally, *I. batatas* contains specific phytochemicals, such as anthocyanins and phenolic compounds, which may benefit lipid metabolism by modulating enzymes involved in lipid production and transport [28]. These findings align with the broader framework of dietary patterns, where consumption of fruits, vegetables, and whole grains has been shown to alter lipid profiles through various biochemical pathways [25, 26].

The findings of this review are broadly consistent with those reported by Naomi et al. [6], who described *I. batatas* as a versatile plant with potential therapeutic relevance. In their study, *I. batatas* administration was associated with improvements in hyperglycemia, with effects greater than those observed for Diabinese, a commonly used antidiabetic agent. After 8 weeks of intervention, improvements in pancreatic *β*‐cell function, reductions in lipid levels, attenuation of insulin resistance, and a lower GI were observed [6].

Although the underlying mechanisms are biologically plausible, the clinical significance of the observed effect sizes remains uncertain. While the pooled analyses demonstrated statistically significant improvements in fasting glucose and lipid parameters, the magnitude of these effects appears modest when compared to commonly used clinical thresholds and established therapeutic interventions. Likewise, although the lipid profile changes were generally favorable, their clinical relevance depends on baseline dyslipidemia severity, the durability of effects over time, and their translatability from preclinical models to humans.

Given that all included studies were conducted exclusively in rat models, these findings should primarily be interpreted as evidence of potential biological efficacy, warranting further confirmation in well‐designed human studies with adequate statistical power and clinically interpretable endpoints.

## 4. Conclusion

Supplementation with *I. batatas* was associated with reductions in FBG and improvements in lipid parameters in rat models of metabolic disturbance. These findings suggest potential biological effects, but they should be regarded with caution, as clinical therapeutic effects remain unconfirmed.

The mechanisms behind these effects are likely related to the low GI, high fiber content, and bioactive compounds in *I. batatas*, which may counteract the effects of high‐glycemic foods and improve dyslipidemia. However, the magnitude of these effects appears modest, and their clinical relevance in humans remains uncertain due to substantial heterogeneity and the use of standardized effect measures.

Future well‐designed, adequately powered randomized controlled trials in human populations are needed to determine whether these changes translate into clinically meaningful reductions in cardiometabolic risk and improvements in cardiovascular health.

## 5. Limitations

The number of articles meeting the inclusion criteria in this meta‐analysis was limited (*n* = 10), potentially affecting the statistical power of the analysis. There was considerable heterogeneity across studies, reflected by variations in animal models, intervention types, dosages, and treatment durations, which may have contributed to the variability in pooled effects. All included studies were conducted in animal models, limiting the generalizability of the findings to human populations. Therefore, these results should be regarded as evidence of the potential biological effects of *I. batatas* in experimental models, not as direct therapeutic effects in humans.

Despite these limitations, this meta‐analysis demonstrated an association between *I. batatas* administration and significant improvements in glycemic and lipid parameters. Future research should include more studies and well‐designed controlled clinical trials in humans to improve the generalizability of the findings and strengthen clinical evidence regarding the effects of *I. batatas* on metabolic parameters.

## Author Contributions

Hapsari Sulistya Kusuma, Mohammad Sulchan, Heri‐Nugroho H.S., Suhartono Suhartono, Diana Nur Afifah, Endang Mahati, Arimi Fitri Mat Ludin, and Adriyan Pramono conceptualized the study. Hapsari Sulistya Kusuma and Adriyan Pramono performed keyword screening, data extraction, and meta‐analysis. All authors participated in data interpretation. Hapsari Sulistya Kusuma and Adriyan Pramono drafted the manuscript, with critical revisions by all authors.

## Funding

This work was supported by Universitas Diponegoro (grant number 222‐234/UN7.D2/PP/IV/2025).

## Conflicts of Interest

All authors declare no competing interests. The corresponding author is supported by Universitas Diponegoro; however, the agency was not involved in the conceptualization, writing, or submission of the manuscript.

## Supporting Information

Additional supporting information can be found online in the Supporting Information section.

## Supporting information


**Supporting Information 1** Figure S1: Subanalysis on the effect of *Ipomoea batatas* leaves and roots on fasting blood glucose.

**Supporting Information 2** File S1: PRISMA 2020 checklist.

**Supporting Information 3** File S2: PRISMA 2020 abstract checklist.

**Supporting Information 4** Table S1: Full electronic search strategy for PubMed.

## Data Availability

The data supporting the findings of this study are available in the references within the article.

## References

[1] Pramono A. , Vitamin D in Insulin Sensitivity and Obesity: Fact or Fiction?, 2020, Doctoral Thesis Maastricht University, Maastricht, 10.26481/dis.20200828ap.

[2] Utari A. , Maududi M. S. , Kusumawati N. R. D. , and Mexitalia M. , Effects of Low Glycemic Index Diet on Insulin Resistance Among Obese Adolescent With Non-Alcoholic Fatty Liver Disease: A Randomized Controlled Trial, Medical Journal of Indonesia. (2019) 28, no. 2, 123–128, 10.13181/mji.v28i2.2496.

[3] Houston T. , Sweet Potato Glycemic Index (46 & 88) Gestation. Diabet, 2023, August 18, 2023 https://thegestationaldiabetic.com/sweet-potato-glycemic-index/.

[4] Kementrian Kesehatan Republik Indonesia , Tabel Komposisi Pangan Indonesia, 2018, Kementrian Kesehatan RI, Jakarta.

[5] Nayar S. and Madhu S. , Glycemic Index of Wheat and Rice Are Similar When Consumed as Part of a North Indian Mixed Meal, Indian Journal of Endocrinology and Metabolism. (2020) 24, no. 3, 251–255, 10.4103/ijem.IJEM_4_20, 33083264. 33083264 PMC7539032

[6] Naomi R. , Bahari H. , Yazid M. D. , Othman F. , Zakaria Z. A. , and Hussain M. K. , Potential Effects of Sweet Potato (*Ipomoea batatas*) in Hyperglycemia and Dyslipidemia—A Systematic Review in Diabetic Retinopathy Context, International Journal of Molecular Sciences. (2021) 22, no. 19, 10816, 10.3390/ijms221910816, 34639164. 34639164 PMC8509747

[7] Pollock D. , Davies E. L. , Peters M. D. J. , Tricco A. C. , Alexander L. , McInerney P. , Godfrey C. M. , Khalil H. , and Munn Z. , Undertaking a Scoping Review: A Practical Guide for Nursing and Midwifery Students, Clinicians, Researchers, and Academics, Journal of Advanced Nursing. (2021) 77, no. 4, 2102–2113. 33543511 10.1111/jan.14743 PMC8049063

[8] Aromataris E. , Fernandez R. , Godfrey C. M. , Holly C. , Khalil H. , and Tungpunkom P. , Summarizing Systematic Reviews, International Journal of Evidence-Based Healthcare. (2015) 13, no. 3, 132–140. 26360830 10.1097/XEB.0000000000000055

[9] Page M. J. , McKenzie J. E. , Bossuyt P. M. , Boutron I. , Hoffmann T. C. , Mulrow C. D. , Shamseer L. , Tetzlaff J. M. , Akl E. A. , Brennan S. E. , Chou R. , Glanville J. , Grimshaw J. M. , Hróbjartsson A. , Lalu M. M. , Li T. , Loder E. W. , Mayo-Wilson E. , McDonald S. et al., The PRISMA 2020 Statement: An Updated Guideline for Reporting Systematic Reviews, BMJ. (2021) 372, 10.1136/bmj.n71. PMC8005924 33782057

[10] Hisamuddin A. S. B. , Naomi R. , Manan K. A. B. , Bahari H. , Othman F. , Embong H. , Ismail A. , Ahmed Q. U. , Jumidil S. H. , Hussain M. K. , and Zakaria Z. A. , The Role of Lutein-Rich Purple Sweet Potato Leaf Extract on the Amelioration of Diabetic Retinopathy in Streptozotocin-Induced Sprague–Dawley Rats, Frontiers in Pharmacology. (2023) 14, 1175907, 10.3389/fphar.2023.1175907, 37274105. 37274105 PMC10232805

[11] Luo D. , Mu T. , and Sun H. , Sweet Potato (Ipomoea Batatas L.) Leaf Polyphenols Ameliorate Hyperglycemia in Type 2 Diabetes Mellitus Mice, Food & Function. (2021) 12, no. 9, 4117–4131, 10.1039/D0FO02733B. 33977940

[12] Novrial D. , Soebowo S. , and Widjojo P. , Protective Effect of *Ipomoea batatas* L Leaves Extract on Histology of Pancreatic Langerhans Islet and Beta Cell Insulin Expression of Rats Induced by Streptozotocin, Molekul. (2020) 15, no. 1, 48–55, 10.20884/1.jm.2020.15.1.563.

[13] Li F. , Li Q. , Gao D. , and Peng Y. , The Optimal Extraction Parameters and Anti-Diabetic Activity of Flavonoids From *Ipomoea batatas* Leaf, African Journal of Traditional, Complementary and Alternative Medicines. (2009) 6, no. 2, 195–202, 10.4314/ajtcam.v6i2.57091, 20209012. PMC2816561 20209012

[14] Ntchapda F. , Tchatchouang F. C. , Miaffo D. , Maidadi B. , Vecchio L. , Talla R. E. , Bonabe C. , Seke Etet P. F. , and Dimo T. , Hypolipidemic and Anti-Atherosclerogenic Effects of Aqueous Extract of *Ipomoea batatas* Leaves in Diet-Induced Hypercholesterolemic Rats, Journal of Integrative Medicine. (2021) 19, no. 3, 243–250, 10.1016/j.joim.2021.02.002, 33775599. 33775599

[15] Jawi I. M. , Yasa I. W. P. S. , and Widhiantara I. G. , Evaluation of the Antiatherogenic Potential of Purple Sweet Potato (*Ipomoea batatas* L.) Extracts in Wistar Rats Exposed to a High-Cholesterol Diet, Journal of Applied Pharmaceutical Science. (2024) 14, no. 2, 152–160, 10.7324/JAPS.2024.115259.

[16] I′tishom R. , Wafa I. , Budi D. S. , and Pratama N. , Oral Delivery of Purple Sweet Potato (*Ipomoea batatas* L.) Extract-Loaded Carboxymethyl Chitosan and Alginate Nanocapsule in Streptozotocininduced Diabetic Mice, Indian Journal of Pharmaceutical Education and Research. (2021) 55, no. 3, 709–714, 10.5530/ijper.55.3.143.

[17] Nagamine R. , Ueno S. , Tsubata M. , Yamaguchi K. , Takagaki K. , Hira T. , Hara H. , and Tsuda T. , Dietary Sweet Potato (*Ipomoea batatas* L.) Leaf Extract Attenuates Hyperglycaemia by Enhancing the Secretion of Glucagon-Like Peptide-1 (GLP-1), Food & Function. (2014) 5, no. 9, 2309–2316, 10.1039/c4fo00032c, 25066255. 25066255

[18] Subawa A. A. N. , Yasa I. W. P. S. , Jawi I. M. , and Mahendra A. N. , Antioxidant and Hypolipidemic Effects of *Ipomoea batatas* L and *Pandanus conoideus* Lam Combination on Rats Fed With High Cholesterol Diet, Open Access Macedonian Journal of Medical Sciences. (2021) 9, A, 473–476, 10.3889/oamjms.2021.6390.

[19] Mi W. , Hu Z. , Zhao S. , Wang W. , Lian W. , Lu P. , and Shi T. , Purple Sweet Potato Anthocyanins Normalize the Blood Glucose Concentration and Restore the Gut Microbiota in Mice With Type 2 Diabetes Mellitus, Heliyon. (2024) 10, no. 11, e31784, 10.1016/j.heliyon.2024.e31784, 38845993. 38845993 PMC11153189

[20] Ojo O. , Ojo O. O. , Adebowale F. , and Wang X.-H. , The Effect of Dietary Glycaemic Index on Glycaemia in Patients With Type 2 Diabetes: A Systematic Review and Meta-Analysis of Randomized Controlled Trials, Nutrients. (2018) 10, no. 3, 10.3390/nu10030373, 29562676. PMC5872791 29562676

[21] Ojo O. , Ojo O. O. , Wang X.-H. , and Adegboye A. R. A. , The Effects of a Low GI Diet on Cardiometabolic and Inflammatory Parameters in Patients With Type 2 and Gestational Diabetes: A Systematic Review and Meta-Analysis of Randomised Controlled Trials, Nutrients. (2019) 11, no. 7, 10.3390/nu11071584, 31336986. PMC6683080 31336986

[22] Wang Q. , Xia W. , Zhao Z. , and Zhang H. , Effects Comparison Between Low Glycemic Index Diets and High Glycemic Index Diets on HbA1c and Fructosamine for Patients With Diabetes: A Systematic Review and Meta-Analysis, Primary Care Diabetes. (2015) 9, no. 5, 362–369, 10.1016/j.pcd.2014.10.008, 25524422. 25524422

[23] Mao T. , Huang F. , Zhu X. , Wei D. , and Chen L. , Effects of Dietary Fiber on Glycemic Control and Insulin Sensitivity in Patients with Type 2 Diabetes: A Systematic Review and Meta-Analysis, Journal of Functional Foods. (2021) 82, 10.1016/j.jff.2021.104500.

[24] Livesey G. , Taylor R. , Livesey H. F. , Buyken A. E. , Jenkins D. J. A. , Augustin L. S. A. , Sievenpiper J. L. , Barclay A. W. , Liu S. , Wolever T. M. S. , Willett W. C. , Brighenti F. , Salas-Salvadó J. , Björck I. , Rizkalla S. W. , Riccardi G. , Vecchia C. L. , Ceriello A. , Trichopoulou A. , Poli A. , Astrup A. , Kendall C. W. C. , Ha M.-A. , Baer-Sinnott S. , and Brand-Miller J. C. , Dietary Glycemic Index and Load and the Risk of Type 2 Diabetes: Assessment of Causal Relations, Nutrients. (2019) 11, no. 6, 10.3390/nu11061436, 31242690. PMC6628270 31242690

[25] Fabiani R. , Naldini G. , and Chiavarini M. , Dietary Patterns and Metabolic Syndrome in Adult Subjects: A Systematic Review and Meta-Analysis, Nutrients. (2019) 11, no. 9, 10.3390/nu11092056, 31480732. PMC6770202 31480732

[26] Lonnie M. , Laurie I. , Myers M. , Horgan G. , Russell W. , and Johnstone A. , Exploring Health-Promoting Attributes of Plant Proteins as a Functional Ingredient for the Food Sector: A Systematic Review of Human Interventional Studies, Nutrients. (2020) 12, no. 8, 10.3390/nu12082291, 32751677. PMC7468935 32751677

[27] Forsberg M. , Forsberg S. , Edman G. , and Hojer J. , No Support for Lipid Rescue in Oral Poisoning: A Systematic Review and Analysis of 160 Published Cases, Human & Experimental Toxicology. (2017) 36, no. 5, 461–466, 10.1177/0960327116679715, 27885103. 27885103

[28] Chen G.-T. , Lu Y. , Yang M. , Li J.-L. , and Fan B.-Y. , Medicinal Uses, Pharmacology, and Phytochemistry of *Convolvulaceae* Plants With Central Nervous System Efficacies: A Systematic Review, Phytotherapy Research. (2018) 32, no. 5, 823–864, 10.1002/ptr.6031, 29356185. 29356185

[29] Pineda E. , Bascunan J. , and Sassi F. , Improving the School Food Environment for the Prevention of Childhood Obesity: What Works and What Doesn’t, Obesity Reviews. (2021) 22, no. 2, e13176, 10.1111/obr.13176, 33462933. 33462933

