# Supplementary material for: The Effect of Ipomoea batatas on Glycemic Control and Lipid Profiles in Animal Models: A Systematic Review and Meta‐Analysis
Source: Int J Food Sci. 2026 Jun 15;2026:1713161. doi: 10.1155/ijfo/1713161 (PMC13266408; doi:10.1155/ijfo/1713161)

**Supplementary Material**

**Supplementary figure of a sub-analysis of sweet potato leaf vs root on fasting blood glucose (FBG)**

**Supplementary Figure 1. Sub-analysis on the effect of *Ipomoea batatas* leaves and roots on Fasting Blood Glucose**


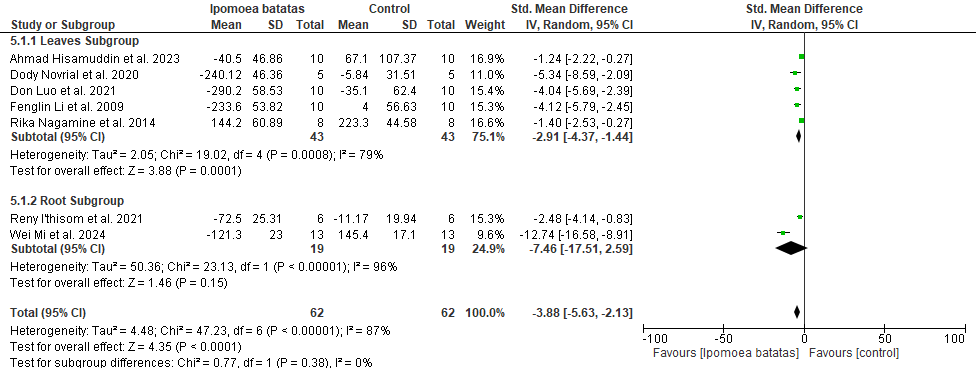

Supplement: Supplementary file 1 — Supporting Information 1 Figure S1: Subanalysis on the effect of Ipomoea batatas leaves and roots on fasting blood glucose. [file IJFO-2026-1713161-s001.docx]
