# Supplementary material for: The Effect of Ipomoea batatas on Glycemic Control and Lipid Profiles in Animal Models: A Systematic Review and Meta‐Analysis
Source: Int J Food Sci. 2026 Jun 15;2026:1713161. doi: 10.1155/ijfo/1713161 (PMC13266408; doi:10.1155/ijfo/1713161)
Supplement: Supplementary file 4 — Supporting Information 4 Table S1: Full electronic search strategy for PubMed. [file IJFO-2026-1713161-s004.docx]

**Supplementary Table S1. Full Electronic Search Strategy for PubMed**

| Database | : | PubMed |
| --- | --- | --- |
| Date of search | : | 21 March 2025 |
| Time range | : | 1 January 2000 - 21 March 2025 |
| Language restriction | : | English |
| Step Search strategy |  |  |
| Step 1 | : | Query:  "Ipomoea batatas"[Title/Abstract] OR "sweet potato"[Title/Abstract] OR "sweet potato extract"[Title/Abstract] |
| Step 2 | : | Query:  "type 2 diabetes"[Title/Abstract] OR "blood glucose"[Title/Abstract] OR "insulin resistance"[Title/Abstract] OR "hyperglycemia"[Title/Abstract] OR "glycemic control"[Title/Abstract] |
| Step 3 | : | Query:  "lipid metabolism"[Title/Abstract] OR "lipid profile"[Title/Abstract] OR "cholesterol"[Title/Abstract] OR "triglycerides"[Title/Abstract] OR "dyslipidemia"[Title/Abstract] |
| Step 4 | : | Query:  "animal study"[Title/Abstract] OR "animal model"[Title/Abstract] OR "in vivo"[Title/Abstract] OR "rodent model"[Title/Abstract] OR "experimental model"[Title/Abstract] |
| Step 5 | : | #1 AND #2 AND #3 AND #4 |
| Final combined search string |  | ("Ipomoea batatas"[Title/Abstract] OR "sweet potato"[Title/Abstract] OR "sweet potato extract"[Title/Abstract]) AND ("type 2 diabetes"[Title/Abstract] OR "blood glucose"[Title/Abstract] OR "insulin resistance"[Title/Abstract] OR "hyperglycemia"[Title/Abstract] OR "glycemic control"[Title/Abstract]) AND ("lipid metabolism"[Title/Abstract] OR "lipid profile"[Title/Abstract] OR "cholesterol"[Title/Abstract] OR "triglycerides"[Title/Abstract] OR "dyslipidemia"[Title/Abstract]) AND ("animal study"[Title/Abstract] OR "animal model"[Title/Abstract] OR "in vivo"[Title/Abstract] OR "rodent model"[Title/Abstract] OR "experimental model"[Title/Abstract]) |
| Filters | : | English; Publication date from 2000/01/01 to 2025/03/21 |
